# The Weighting of Cues to Upright Following Stroke With and Without a History of Pushing

**DOI:** 10.1017/cjn.2017.297

**Published:** 2018-06-21

**Authors:** Lindsey E. Fraser, Avril Mansfield, Laurence R. Harris, Daniel M. Merino, Svetlana Knorr, Jennifer L. Campos

**Affiliations:** 1 Toronto Rehabilitation Institute, University Health Network, Toronto, ON, Canada; 2 Heart and Stroke Foundation Canadian Partnership for Stroke Recovery, Toronto, ON, Canada; 3 Sunnybrook Research Institute, Toronto, ON, Canada; 4 Department of Psychology, University of Toronto, Toronto, ON, Canada; 5 Centre for Vision Research, York University, Toronto, ON, Canada; 6 Department of Physical Therapy, University of Toronto, Toronto, ON, Canada

**Keywords:** Pushing, Stroke, Vision, Body, Graviception

## Abstract

***Objective:*** Perceived upright depends on three main factors: vision, graviception, and the internal representation of the long axis of the body. We assessed the relative contributions of these factors in individuals with sub-acute and chronic stroke and controls using a novel tool; the Oriented Character Recognition Test (OCHART). We also considered whether individuals who displayed active pushing or had a history of pushing behaviours had different weightings than those with no signs of pushing. ***Method:*** Three participants experienced a stroke <3 months before the experiment: one with active pushing. In total, 14 participants experienced a stroke >6 months prior: eight with a history of pushing. In total, 12 participants served as healthy aged-matched controls. Visual and graviceptive cues were dissociated by orienting the visual background left, right, or upright relative to the body, or by orienting the body left, right, or upright relative to gravity. A three-vector model was used to quantify the weightings of vision, graviception, and the body to the perceptual upright. ***Results:*** The control group showed weightings of 13% vision, 25% graviception, and 62% body. Some individuals with stroke showed a similar pattern; others, particularly those with recent stroke, showed different patterns, for example, being unaffected by one of the three factors. The participant with active pushing behaviour displayed an ipsilesional perceptual bias (>30°) and was not affected by visual cues to upright. ***Conclusion:*** The results of OCHART may be used to quantify the weightings of multisensory inputs in individuals post-stroke and may help characterize perceptual sources of pushing behaviours.

## RÉSUMÉ

**Le poids des repères impliqués dans la perception de la verticalité chez des patients atteints d’un AVC ayant ou non des antécédents de latéropulsion.** ***Objectif:*** La perception de la verticalité dépend de trois principaux facteurs: la vision, la perception de la gravité (*graviception*) et la représentation interne de l’axe longitudinal du corps. Nous avons cherché à évaluer l’impact relatif de ces facteurs chez des individus atteints d’AVC subaigus et chroniques et des sujets d’un groupe témoin en faisant appel à un outil innovateur: le *Oriented Character Recognition Test* (OCHART). Nous avons aussi cherché à savoir si les patients montrant des signes de latéropulsion active ou ayant des antécédents de latéropulsion accordaient un poids différent aux repères de la perception de la verticalité, et ce, en comparaison avec ceux ne montrant aucun signe de latéropulsion. ***Méthodes:*** Trois participants avaient été atteints d’un AVC moins de 3 mois avant la réalisation de cette expérience, dont un montrant des signes de latéropulsion active. Quatorze d’entre eux avaient été atteints d’un AVC plus de 6 mois avant cette expérience, dont huit ayant des antécédents de latéropulsion. Au total, douze participants en santé ont été jumelés selon l’âge et ont servi de témoins. Les repères visuels et graviceptuels avaient été dissociés en orientant l’arrière-plan visuel vers la gauche, la droite ou le haut par rapport aux corps des participants; ou bien en orientant leurs corps vers la gauche, la droite ou le haut par rapport à la gravité. Un modèle doté de trois vecteurs a également été utilisé pour quantifier le poids de la vision, de la graviception et du corps dans la perception de la verticalité. ***Résultats:*** Les sujets du groupe témoin ont montré un poids de 13%, 25% et 62% en ce qui regarde respectivement la vision, la graviception et le corps. Certains patients atteints d’AVC ont montré un profil similaire ; d’autres, particulièrement ceux récemment victimes d’un AVC, ont présenté des profils différents, n’étant pas, par exemple, affectés par un de ces trois facteurs. Le participant montrant des signes de latéropulsion active a présenté quant à lui un biais perceptuel ipsilésionnel (>30°) et n’a pas été affecté par les repères visuels de la perception de la verticalité. ***Conclusion:*** Il est possible d’utiliser les résultats de l’OCHART pour quantifier, à la suite d’un AVC, le poids respectif des inputs multi-sensoriels chez des individus. Cet outil pourrait également permettre de mieux caractériser les causes perceptuelles liées à la latéropulsion.

The subjective perception of upright is derived from three distinct factors: vision (e.g., visual cues from the horizon; objects resting on other objects);^1^ graviception (the direction of gravity as detected by the vestibular system, pressure on the skin and other somatic receptors in the body);^2^ ^,^ ^3^ and the internal representation of the long axis of the body.^3^ The perception of upright is traditionally assessed by asking participants to align their bodies with gravitational vertical (subjective postural vertical [SPV]), or to align a visual stimulus such as a luminous vertical line with gravitational vertical (subjective visual vertical [SVV]).^4^ ^-^ ^8^ The goal of these tasks is often to detect deviations in the perception of upright in individuals with neurologic injury compared those without. The SPV and SVV tasks primarily target perceived upright as informed by somesthetic and vestibular contributions respectively^1^ ^,^ ^9^ and as such do not directly consider how these and other inputs (e.g., visual) are weighted when they are all present. In order to determine the unique contributions of specific sensory inputs, sensory-conflict paradigms have been used to present observers with information about multiple perceptual uprights (PUs) at the same time; for instance, by tilting the body (which separates the longitudinal body axis from vestibular and somesthetic graviceptive cues) or tilting the visual scene (which separates visual from graviceptive and/or body axis cues). Dyde et al.^1^ used the sensory-conflict method to determine the contributions of vision, graviception, and body cues to the SVV in healthy adults. They found an average weighting of 15% for visual cues, 77% for graviceptive cues, and 8% for body cues. That is, in the SVV task, graviceptive cues were weighted much higher than either visual cues or the body. However, this higher weighting of graviceptive cues may be due, in part, to the explicit goals of the SVV task; that is, to “judge the orientation of a line relative to *gravitational* vertical”.^4^ ^,^ ^6^ ^,^ ^10^ ^,^ ^11^ When attempting to characterize the relative weights of different sensory inputs to perception of upright under typical everyday conditions, a more neutral task that does not prioritize a specific frame of reference may be preferred. In other words, a task that does not ask an observer to judge their orientation “relative to gravity” specifically, may be better suited for quantifying multisensory contributions to the perception of upright rather than tasks that target the ability of an observer to use one category of sensory inputs over others.

In order to serve the goal of providing a more “sensory-neutral” assessment of perceived upright, the Oriented Character Recognition Test (OCHART^1^) was developed and has been used extensively in healthy younger adults^1^ ^,^ ^12^ ^-^ ^17^ with evidence of good test-retest reliability.^18^ In this task participants are shown a character (“p”) at various orientations (e.g., 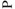) and are asked to report its identity (either “p” or “d”). The character’s identity is ambiguous unless the participant makes an assumption about its orientation. The test yields a score that reflects the orientation at which the character is most easily recognized, defined as the “perceptual upright” (PU).^1^ The Oriented Character Recognition Test does not require explicit cognitive judgements about self-orientation or verticality, and does not emphasize any particular sensory input over another. Previous results obtained when implementing the OCHART within the sensory-conflict paradigm described above showed average weightings of 25% for visual cues; 21% for graviceptive cues; and 54% for the internal representation of the body axis in healthy adults.^1^ Thus, there is a more equal distribution of weightings seen when performing the OCHART compared with when performing the SVV task.

Understanding whether and how specific sources of information are being used to inform perceptual upright can be particularly important when misperceptions occur, such as in the case of individuals with neurologic injury. Of particular interest is post-stroke “pushing” behaviour, which is characterized by a postural lean to the contralesional side and resistance to being oriented upright.^19^ Individuals with pushing behaviours show a marked bias in SPV such that they feel upright when their body is tilted about 20° to the side in the roll axis.^7^ The SVV literature is more mixed, with some studies reporting a bias concurrent with active pushing (AP) behaviours^6^ and a history of pushing (HP),^5^ and others reporting more variability, but no systematic bias compared with healthy controls.^4^ It has been argued that pushers’ large SPV biases indicate a compromised internal estimate of the body midline. In contrast, the smaller or absent SVV errors in this population suggest vestibular and visual perception may be spared.^20^ ^-^ ^22^ Thus, pushing is characterized as a result of an impairment in the network for body perception and postural control.^11^

What remains unknown is the extent to which different sources of sensory inputs are weighted by individuals with AP behaviour or a HP. As such, the calculated weights from the OCHART may provide unique and valuable insights into the multisensory processes underlying the perception of upright following stroke and into the perceptual source(s) of error contributing to pushing behaviour at the individual person level. Indeed, understanding whether an observer *is able to* use a specific sensory input to judge upright is not the same as understanding the *extent to which* they actually use this input and integrate it centrally with other available and often redundant inputs. Given the heterogeneity of the constellation of post-stroke impairments, the sites of infarct, and the likely presence of other co-morbidities, the capacity to model the weightings used by each individual and to look for commonalities across groups of participants with similar symptoms is particularly valuable. For instance, determining that the percepts of an individual with pushing behaviours are not at all influenced by visual cues to upright and are primarily driven by graviceptive cues to upright could have important implications for the rehabilitative strategies chosen by therapists.

Therefore, in this study we implemented a sensory-conflict paradigm and employed the OCHART task in individuals at sub-acute and chronic stages of stroke with and without AP behaviour or a HP behaviour and compared their performance to a group of healthy, age-matched controls. Our main objectives were to determine whether (1) individuals with a history of stroke demonstrate a different pattern of weightings (visual, graviceptive, and body) compared with age-matched controls, and (2) whether individuals who displayed pushing behaviour following stroke have different sensory weights compared with those who show no signs of pushing. In line with previous research showing that individuals with stroke represent the orientation of their body axis with less precision than controls,^22^ we hypothesized that those with stroke would show a higher relative weighting of visual and graviceptive cues compared with controls. We also predicted that among those with stroke, individuals with AP and those with a HP would show the lowest weighting of the body axis. We also predicted that individuals with AP may present a bias in their perceptual upright even when all available cues were in agreement, consistent with the theory that pushing may reflect a mislocalization of the body midline with respect to gravity.^11^ ^,^ ^20^

## Material and Methods

### Participants

#### Control Participants

In total, 12 healthy older adults (six men, six women; mean age 65±7 years: roughly age-matched to the stroke participant samples) were recruited from a pre-existing Toronto Rehabilitation Institute volunteer pool, by posters, or by word of mouth.

#### Sub-Acute Stroke Participants

Three individuals who had experienced a single stroke event <3 months before testing (two men, one woman, mean age 70±8 years) were recruited from the in-patient stroke rehabilitation unit at the Toronto Rehabilitation Institute. One of these patients was classified as having “AP behaviour” by his physiotherapist, who also reported that the patient leaned to his contralesional (right) side, although the Scale for Contraversive Pushing (SCP^23^) could not be completed because of the patient’s severe postural impairment. The remaining two sub-acute participants (no active pushing, NAP) displayed no evidence of AP behaviour (SCP=0). Please refer to Table 1 for specific details of lesion type and location for all stroke participants.

#### Chronic Stroke Participants

In total, 14 individuals who had experienced a single stroke event >6 months before testing were recruited (six men, eight women, mean age 66±10 years) from a pool of participants in an ongoing longitudinal study on stroke recovery and a pre-existing Toronto Rehabilitation Institute database of former patients who had consented to be contacted for research. Of these 14 individuals, eight had a HP behaviours as identified by a score of ≥1 on item C of the SCP,^24^ “resists correction”, immediately following their stroke, or by a record of pushing behaviour following stroke as indicated in their hospital charts by trained clinicians. The HP group all scored 0 on the SCP (standing and sitting) at the time of the study. The other six participants in the stroke group had no recorded history of pushing (NHP). Specific details of stroke location and type are reported in Table 1.

All participants (stroke and controls) reported no other incidental neurological or musculoskeletal conditions likely to impair balance and had no history of vestibular disorders (e.g., vertigo, chronic dizziness). Participants all scored at least 20/50 on a Snellen eye exam, and could communicate clearly in English. Of those who spoke English as a second language, we included only those whose first language used a Latin-derived alphabet to avoid biases in the ease of identifying a p versus a d. The Sunnybrook Neglect Assessment Procedure (SNAP) was used to assess the severity of visuo-spatial neglect.^25^ The SNAP is a pen and paper test that includes four items to assess visuo-spatial neglect: copying and drawing, line cancellation, line bisection, and shape cancellation. Control participants scored within the typical range for their age and sex on the Berg Balance Scale,^26^ ^,^ ^27^ a 14-item observational rating scale that provides a measure of functional balance. They also had had no evidence of neurological impairment evidenced by the National Institutes of Health Stroke Scale (NIH SS),^26^ an 11-item scale that provides a gross measure of the effects and severity of stroke. Individual items on the NIH SS evaluate: level of consciousness, gaze, visual field, facial palsy, motor function (arm and leg), ataxia, pain sensation, language, dysarthria, and visual, tactile, spatial, or personal inattention. Participants had normal plantar and palmar cutaneous sensation as measured by monofilaments. Individual characteristics of stroke group participants are listed in Table 1.

**Table 1:** Participants’ details

											Weightings			Biases	
ID	Group	Age (years)	Sex	Time post-stroke (months)	Stroke type	Stroke location	Early SCP score	SNAP score	NIH SS score	BBS score	Body (%)	Vision (%)	Graviception (%)	PU	SVV
Control															
		65 (55, 79)	6 M, 6 F	-	-	-	-	0 (0, 3)	-	56 (54, 56)	62 [41, 84]	13 [6, 19]	25 [5, 45]	−3 [−8, 3]	−1 [−2, 0]
Sub-acute															
A	NAP1	64	M	2	Ischaemic	Right internal capsule and pons	0	0	4	41	83	0	17	−15	−5
B	NAP2	79	F	1	Ischaemic	Right basal ganglia	0	0	3	29	0	17	83	−22	n/a
C	AP	67	M	1	Ischaemic	Left middle cerebral artery/anterior cerebral artery territory	-	0	9	8	57	0	43	34	0
Chronic															
D	NHP	66	F	7	Ischaemic	Left basal ganglia	0	0	2	53	0	42	58	−12	0
E	NHP	49	F	16	Ischaemic	Right pons	-	0	2	55	46	54	0	−5	0
F	NHP	62	F	17	Lacunar	Left internal capsule: posterior limb	-	2	1	56	68	7	25	22	−2
G	NHP	58	M	12	Ischaemic	Right internal capsule	-	7	1	56	84	16	0	−2	−1
H	NHP	52	F	15	Haemorrhagic	Right basal ganglia and thalamus	-	0	2	51	44	19	36	55	1
I	NHP	69	F	11	Ischaemic	Left anterior insula and frontal operculum	-	0	1	49	61	15	24	−9	3
J	HP	80	M	45	Haemorrhagic	Right thalamus	3	0	1	37	45	11	44	1	−3
K	HP	77	M	12	Ischaemic	Left periventricular	-	2	3	41	9	1	91	47	−4
L	HP	66	M	50	Ischaemic	Right parietal and frontal	3	33*	8	26	95	5	0	−2	−1
M	HP	72	M	17	Ischaemic	Right pons	-	3	1	54	84	16	0	−4	−1
N	HP	79	F	16	Ischaemic	Right parietal and internal capsule	5.25	60**	4	37	89	0	11	−48	−13
O	HP	56	M	15	Ischaemic	Right parietal, frontal and temporal	5.75	30*	6	5	1	19	80	−22	−2
P	HP	78	F	16	Ischaemic	Left parietal and frontal	1.25	6*	2	29	28	8	64	19	0
Q	HP	57	F	55	Ischaemic	Right middle cerebral artery territory	-	5*	2	49	3	36	61	−6	−2

SCP=Scale for Contraversive Pushing;^24^ SNAP=Sunnybrook Neglect Assessment Procedure; NIH SS=National Institutes of Health Stroke Scale;^28^ BBS=Berg Balance Scale;^27^ ^,^ ^34^ PU=perceptual upright; SVV=subjective visual vertical; NAP=sub-acute stroke participants with no active pushing; AP=active pusher; NHP=no history of pushing; HP=history of pushing. Sensory weightings in the final three columns are expressed as relative percentages (adding up to 100%). Biases are reported for the Oriented Character Recognition Test (blank background) and SVV (blank background^5^). Biases are in degrees, with negative values indicating leftward deviations (controls) or contralesional deviations (stroke groups) from the body midline. For controls, reported scores are the group mean. Values in round brackets are ranges and values in square brackets are 95% confidence intervals.

* Indicates mild to moderate hemispatial neglect.

** Indicates severe hemispatial neglect.^25^

Participants who had to travel from outside Toronto Rehab to participate in the study received $40 CAD to compensate them for their travel expenses (two visits). All participants provided written consent before their participation. This study was approved by the Toronto Rehabilitation Institute’s Research Ethics Board and was conducted in accordance with the Declaration of Helsinki.

### Apparatus

**Figure 1:**
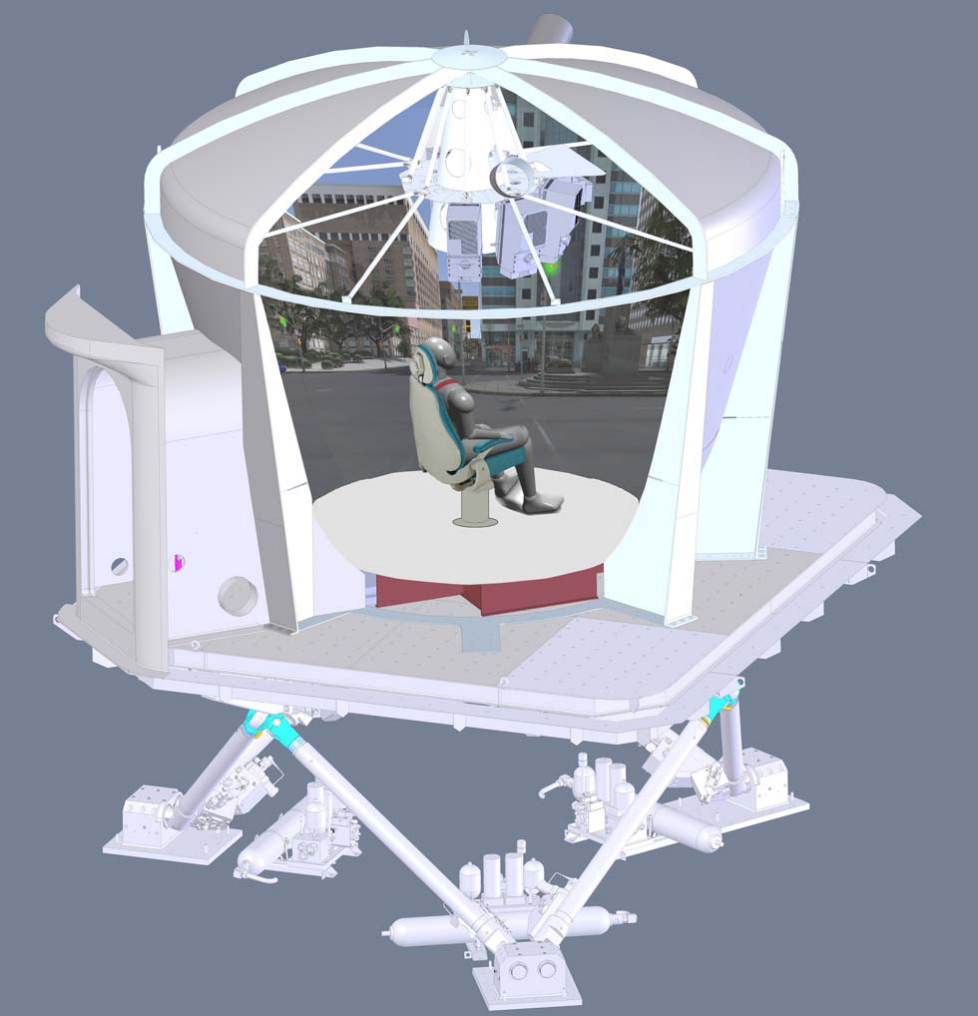
StreetLab on CEAL’s motion platform. See www.idapt.com for more details.

The study was conducted in StreetLab, a Virtual Reality laboratory located within the Challenging Environment Assessment Laboratory at the Toronto Rehabilitation Research Institute (www.idapt.com). StreetLab consists of a high-resolution, 240° horizontal×110° vertical field-of-view curved projection screen and a calibrated six projector system (Eyevis ESP-LED, Eyevis, Reutlingen, Germany) (Figure 1). Participants sat in a chair in the centre of the lab such that the screen occupied their entire field of view. The simulated street scene consisted of a static rendering of a traffic intersection in downtown Toronto, located immediately in front of the Toronto Rehabilitation Institute’s University Centre using a customized OpenScene Graph application. The scene provided strong visual cues to orientation, including buildings, light posts, trees, and street signs. The scene could be presented in any orientation relative to the participant. In this experiment the scene was presented either in an upright configuration, or tilted to the left or right by 112° (these roll angles have been previously shown to shift the PU the greatest amount^1^). For blank screen trials (no visual input) the display was a uniform blue of approximately equal luminance to the average luminance of the city scene. The chair was equipped with footrests and a headrest, and participants were secured to the chair with a seatbelt and to the ceiling of the lab by a safety harness. A stiff neck pillow prevented, as best as possible, movement of the head on the shoulders.

The entire StreetLab module was mounted on top of a 6-DOF hexapod motion platform comprised of six hydraulic actuators, each with a 1.52 m stroke length (HyMotion-11000-6DOF-1524-MK5-SP, Bosch Rexroth B. V., Boxtel, The Netherlands; see Figure 1). This enabled us to physically tilt the participant together with, or separate from the visual display. During the experiment, the platform was oriented at one of three positions: upright, tilted 20° to the left (roll), or to 20° to the right (roll).

### The Subjective Visual Vertical Test

As a baseline measure, all controls and chronic stroke participants completed the SVV task as a point of comparison against the sensory-neutral outcomes provided by the OCHART (some of these data for the HP, NPH, and control groups have been previously presented in Mansfield et al.^5^). In this study, the SVV was performed while upright with eyes open and with no projected visual scene present. A white line subtending 3° of visual angle was projected on the screen. Participants were asked to judge if the line would topple to the left or right. A psychometric function was generated from 30 trials using the adaptive staircase procedure QUEST.^29^ The SVV error was calculated by subtracting the point of subjective equality (i.e., angular bias) of the resulting function from true gravitational upright.^5^.

### The Oriented Character Recognition Test

The Oriented Character Recognition Test probe character (the letter “p”) was rendered in white font and presented in the middle of the screen, subtending ~3° of visual angle. During trials when the city scene was present, the character appeared superimposed on the scene. To assess PU, this character was presented statically at different orientations and participants were asked to report whether they saw a “p” or a “d”. The two points of maximum ambiguity (i.e., the position at which the characters’ identity was reported as “p” or “d” with equal likelihood) were determined using two Bayesian adaptive staircases (QUEST^29^), each of which honed in on one orientation.^a^ The average of the two resulting orientations was calculated as the PU, the orientation at which the character was most easily recognized (see Dyde et al.^1^ for more details on the method of calculating the PU). For each QUEST, the staircase ran for 30 trials, such that each experimental condition required 60 trials.

### Procedure

**Figure 2:**
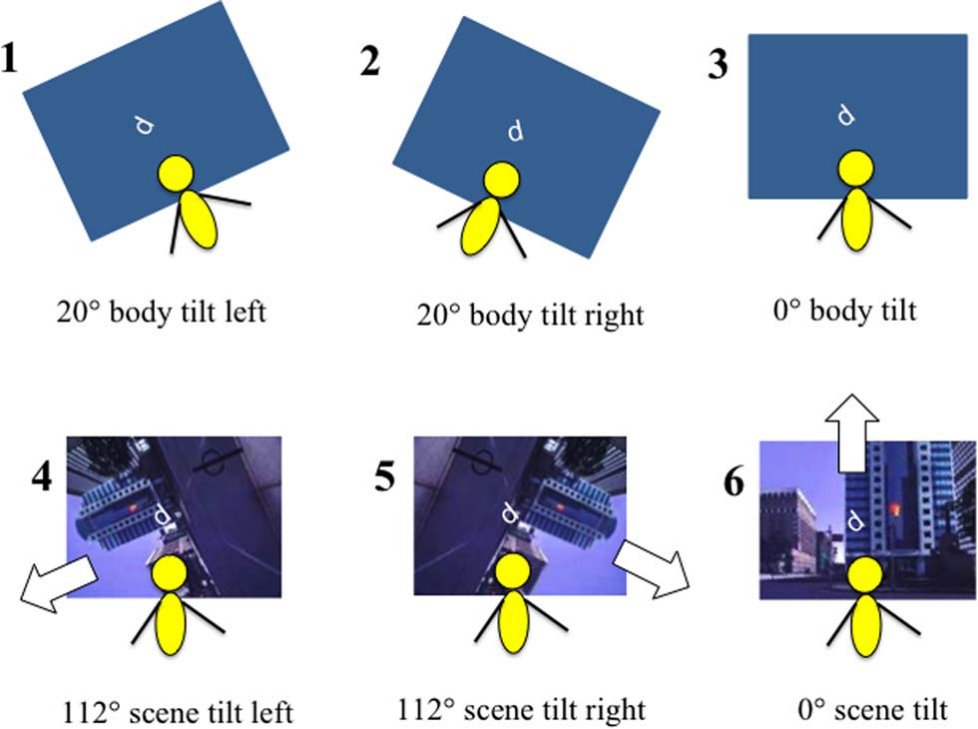
The six stimulus configurations in which the perceptual upright was assessed using Oriented Character Recognition Test (OCHART). Top row: “Body Orientation” conditions. The OCHART was completed with a blank visual background and the platform was tilted 20° to the left (condition 1), right (condition 2), or remained upright (condition 3). Bottom row: “Visual Orientation” conditions. OCHART was performed while sitting physically upright, with a visual background that was tilted 112° to the left (condition 4), right (condition 5), or remained upright (condition 6).

Participants were seated in the testing chair in StreetLab. Each participant completed the SVV task while oriented upright and the OCHART under the six conditions described below. For the OCHART, the platform was moved to the appropriate orientation for that trial (upright, tilted 20° left, or tilted 20° right; Figure 2) and the appropriate scene was projected on the screen (blank, scene upright, scene tilted 112° left, or scene tilted 112° right). The OCHART character appeared and participants were instructed to respond verbally whether it appeared to be a “p” or a “d”, as quickly as possible while still being accurate. The display remained on until the participant had responded. An experimenter within StreetLab, seated behind and out of view of the participant, recorded their answer on a tablet, and the next character then appeared in the next test orientation. On each trial, one of the two QUESTs determined the orientation of the presented character.

#### Body Orientation Conditions (Conditions 1-3)

During the body orientation conditions the room was dark, the visual background was blank, and participants were either physically tilted 20° to the left (condition 1), tilted 20° to the right (condition 2), or upright (condition 3) (see Figure 2).

#### Visual Orientation Conditions (Conditions 3-6)

During the visual orientation conditions, the participants remained oriented in a physically upright position. The visual scene was either blank (condition 3), tilted 112° to the left (condition 4), tilted 112° to the right (condition 5), or was upright (condition 6) (see Figure 2).

The conditions for which the activation of the motion base was required (conditions 1 and 2) were always completed first in a block design, counterbalanced in order across participants, to avoid moving the participant from side to side repeatedly. Conditions 1 and 2 each took ~7 minutes to complete. Subsequently, the motion platform was oriented upright and the remaining four conditions (conditions 3-6) were randomly interleaved (a total of 240 trials), which took ~25 minutes total to complete. Participants were offered a rest break halfway through this final test block, and were permitted to take breaks throughout the test session if requested.

### Convention

For the data presented below, 0° indicates aligned with the body. For analyses comparing the stroke group to controls, negative values indicate a leftward or counter-clockwise bias from the body midline, and positive values indicate a rightward or clockwise bias. For comparisons between HP and NHP groups we coded PU relative to lesion side; negative values indicate contralesional bias and positive values are ipsilesional bias.

### Data Analysis

Participants yielded a single SVV score and a single OCHART PU score per condition. Thus, each participant yielded one SVV score and six PU scores in total. One sub-acute participant (NAP1) did not complete the SVV task due to fatigue. Due to the small number of sub-acute patients in our study, these individuals were not subjected to any group-level statistical analyses. For the PU scores, comparisons were made between: (1) individuals with chronic stroke and age-matched controls; and (2) the HP and NHP chronic stroke participant sub-groups, to see whether tilting the body with respect to gravity or tilting the visual scene would alter PUs differently for the compared groups.

#### Stroke Versus Control Participants

To test participants’ sensitivity to changes in the direction of gravity relative to the body, we subtracted each participant’s PU score in condition 1 (where a higher weighing of graviception would shift PU to the right, towards the gravity-indicated vertical), from the PU score in condition 2 (where graviception would shift PU to the left). This captures the magnitude of the change in PU elicited by tilting the body with respect to gravity, which we refer to as the graviceptive effect. A small graviceptive effect would suggest little sensitivity to graviceptive cues to PU, whereas a large effect would suggest a high sensitivity to graviceptive cues. We compared graviceptive effects in the stroke group to those in the control group using an independent-samples Mann-Whitney *U* test. We predicted that the graviceptive effect for individuals with stroke would be greater than that for controls, meaning that individuals with stroke would place more emphasis on graviceptive cues compared with controls.

Similarly, we measured sensitivity to changes in visual scene orientation by subtracting the PU scores in condition 5 (where a higher weighting of vision would shift PU to the right) from PU scores in condition 4 (where PU would shift to the left). We refer to this as the visual effect. The visual effect was compared between the stroke group and the control group using an independent-samples Mann-Whitney *U* test. We predicted that the visual effect for individuals with stroke would be greater than that for controls in that individuals with stroke would weight visual cues higher than would controls.

#### Comparing Stroke Participant Sub-groups

We sub-divided the individuals with chronic stroke into an HP group and a NHP group to determine whether a HP changes the relative emphasis on graviceptive and visual cues, even after recovery from overt pushing behaviours. Biases were coded with respect to lesion side (negative values indicating a contralesional bias). Following this re-coding, the graviceptive effect and visual effect were calculated. The graviceptive effect was calculated as the PU estimates for the condition during which gravitational upright was to the ipsilesional side of the participant (where a positive shift of the PU would be expected), minus the PU score for the condition during which gravity was to the contralesional side (where a negative shift would be expected). The visual effect was calculated as the PU estimate for the condition during which visually indicated upright was to the ipsilesional side (where a positive shift of the PU would again be expected) minus the PU score in the condition during which visually indicated upright was to the contralesional side (where a negative shift would be expected). The graviceptive effect and visual effect were each compared between stroke participant groups using independent-samples Mann-Whitney *U* tests. It was predicted that both the graviceptive effect and visual effect would be greater for HP compared with NHP participants.

#### Measuring Overall Bias

We compared PU scores from condition 3 (physically upright in the dark) and condition 6 (physically upright with visual cues to upright present) in each group (controls, HP and NHP) to 0° (i.e., “accurate” or zero bias) using one-sample Wilcoxon signed rank tests to determine whether the PU was significantly biased from the orientation indicated by all available inputs (i.e., true upright). A bias in PU in one or both of these conditions would suggest an underlying error in PU even in the presence of multiple congruent sensory inputs specifying upright.

Finally, we fitted each participant’s data, including those of the sub-acute participants, with a three-vector model outlined in Dyde et al.^1^ and described below, to determine the relative weighting of visual, graviceptive, and body inputs to PU for each participant.

## Results

### SVV

**Figure 3:**
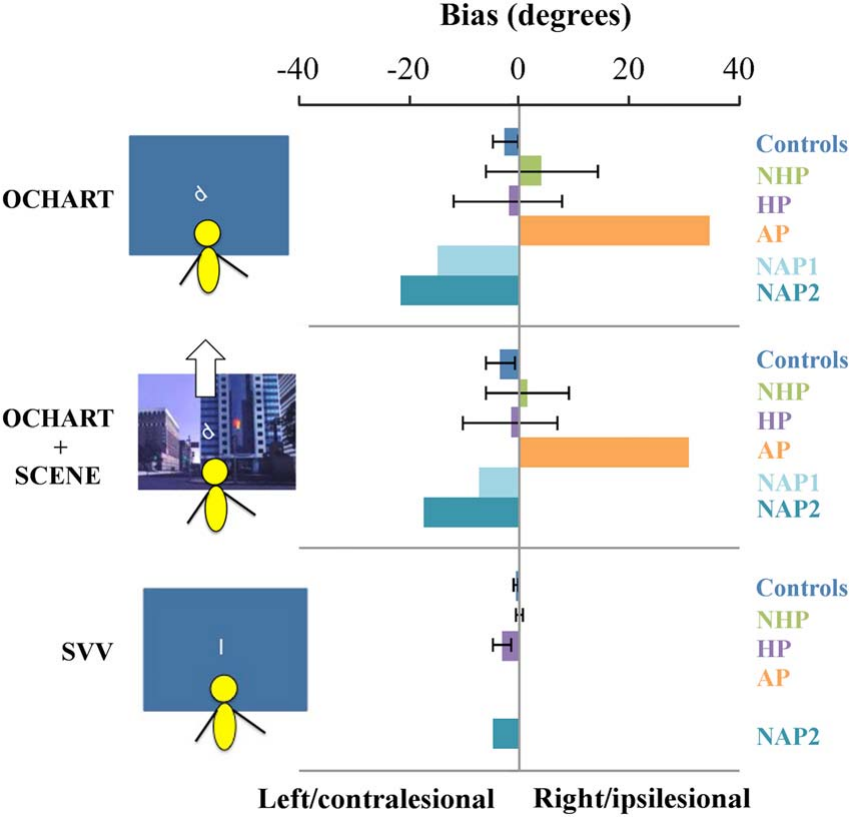
Average bias in perceptual upright in condition 3 (body and graviceptive aligned), and in condition 6 (body, graviceptive, and visuals aligned), and average bias in subjective visual vertical (SVV) response (body and graviceptive aligned) for individuals with chronic stroke with a history of pushing (HP) and with no history of pushing (NHP), the sub-acute participant with active pushing (AP) and those with no active pushing (NAP1, NAP2), and controls. The SVV bias for NAP1 is not shown as the participant did not complete this task. The SVV bias for AP was 0. Note that for controls, negative values indicate a leftward bias from the body midline, whereas for stroke participants negative values indicate a contralesional bias. Error bars are standard error of the mean. OCHART=Oriented Character Recognition Test.

Individual SVV biases are shown in Figure 3. Two of the chronic stroke participants with a HP behaviour (participants O and L) had contralesional biases that were outside the range of the healthy controls (−12.6 and −4°, respectively) and as a group overall, the HP chronic stroke participant group had a significant contralesional bias in SVV (one-sample Wilcoxon signed rank test, *Z*=0, *p*=0.018), but the NHP group did not (*Z*=9.0, *p*=0.69).^5^ Healthy control participants also did not have a significant bias in SVV scores (*Z*=8.5, *p*=0.10). The one NAP participant who completed the SVV task showed a contralesional bias, but the AP showed no bias in their SVV.

### Oriented Character Recognition Test

#### Graviceptive Effect and Visual Effect

**Figure 4:**
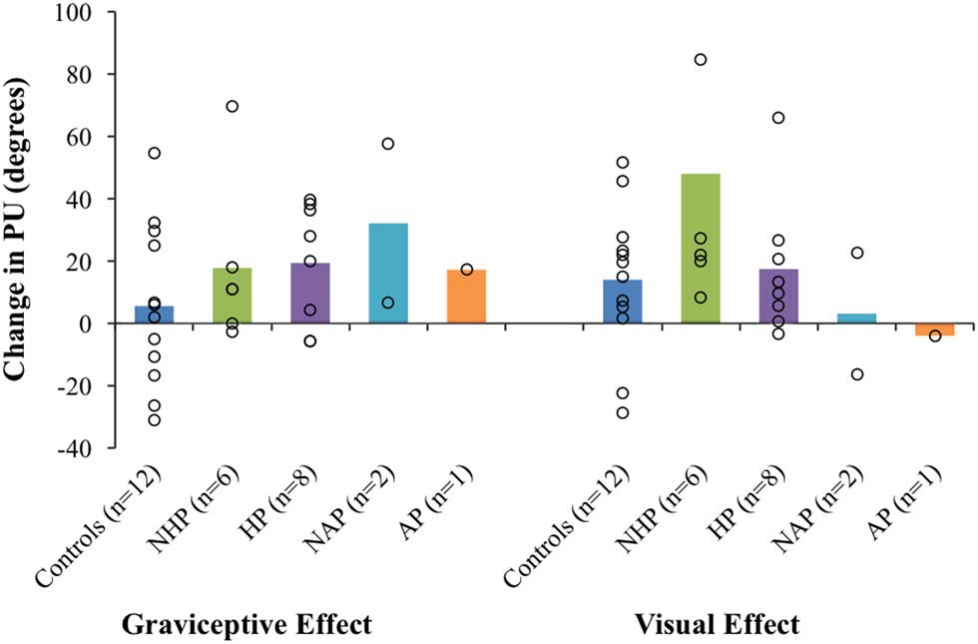
Mean graviceptive effects and visual effects for controls, individuals with chronic stroke (with a history of pushing [HP] and with no history of pushing [NHP]), sub-acute stroke without pushing (no active pushing [NAP]), and the active pusher (AP). Open circles are scores for individuals; bars indicate group averages. Error bars are standard error of the mean. PU=perceptual upright.

Figure 4 shows the mean graviceptive effect and mean visual effect for controls, individuals with chronic stroke (HP and NHP combined), sub-acute non-pushers (NAP1 and NAP2) and the sub-acute AP. Table 2 shows the graviceptive and visual effects for the different groups, including the four sub-acute participants.

**Table 2:** Shift in perceptual upright (PU) caused by tilting the body relative to gravity (graviceptive effect) or tilting the visual scene (visual effect)

Groups	Graviceptive effect (°)	Visual effect (°)
Controls (*n*=12)	6±7	14±7
Chronic		
NHP (*n*=6)	12±6	32±20
HP (*n*=8)	32±15	24±7
Sub-acute		
AP	17	−4
NAP1	7	−16
NAP2	58	23

NHP=no history of pushing; HP=history of pushing; AP=active pusher; NAP=sub-acute stroke participants with no active pushing. Where group means are shown, standard errors are included.

A Mann-Whitney *U* independent-samples test found no significant differences between the graviceptive effects for the chronic stroke participant group (NHP and HP) compared with controls, *U*=110.0, *p*=0.19. A second Mann-Whitney *U* test found no significant differences between the visual effects for the chronic stroke participant group compared with controls, *U*=98.5, *p*=0.46. Similarly, Mann-Whitney *U* tests found no significant differences for the graviceptive effects or the visual effects between HP and NHP sub-groups (graviceptive effects, *U*=32.0, *p*=0.35; visual effects, *U*=28.0, *p*=0.66).

#### Overall Bias

Figure 3 shows the mean PU biases in conditions 3 and 6 (congruent and aligned sensory conditions) for control participants, chronic stroke participants (NHP and HP), and sub-acute stroke participants (AP and NAP). Figure 3 also shows the SVV biases for each group.

One-sample Wilcoxon signed rank tests comparing scores to a median of 0° (no bias) were not significant for PU scores in condition 3 (in the dark with just body and gravity aligned) for controls (*Z*=21, *p*=−0.16). Similarly, there was no difference between PU responses in condition 6 (with body, graviceptive, and visual cues aligned) and 0° for controls (*Z*=53.0, *p* −0.27). There were no significant contra- or ipsilesional biases in these conditions for the PU for either the HP or NHP groups (HP, condition 3, *Z*=13.0, *p*=0.48; HP condition 6, *Z*=15.0, *p*=0.67, *d=*−0.07; NHP condition 3, *Z*=11.0, *p*=0.92; NHP condition 6, *Z*=7.0, *p*=0.46).

#### Modelling the Data

The above analysis of the effects of each sensory manipulation did not reveal any differences at the group level. However, a more precise, customized, evaluation can be achieved by calculating relative sensory input weightings at the individual participant level. This is an important approach given the typical heterogeneity of this population for which traditional group-level analyses are not sensitive enough to characterize unique differences among participants. This approach is also appropriate to use with small sample sizes, such as the sub-acute participant group in the current study for which we do not have enough statistical power to perform inferential statistics.

**Figure 5:**
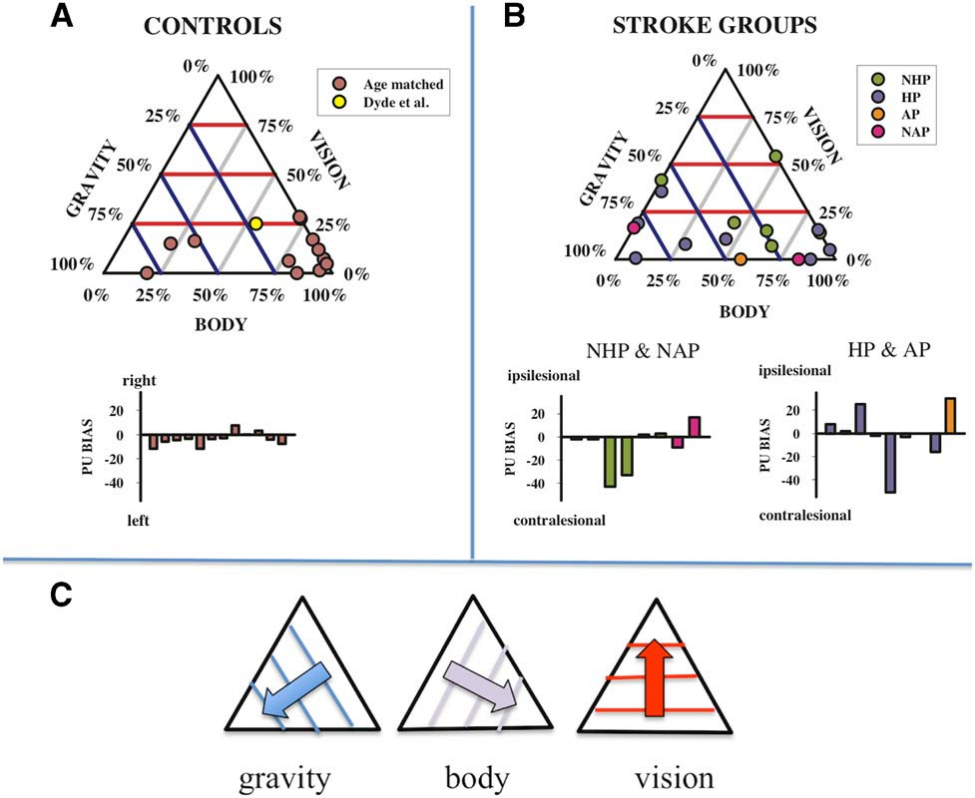
Ternary plots of the relative weightings of the body, graviceptive, and visual cues to perceptual upright (PU) in healthy age-matched controls as well as in reference to previously published data^6^ in healthy younger adults (A), and stroke groups (B) including those with sub-acute stroke and no active pushing (NAP), sub-acute stroke and active pushing (AP), chronic stroke with no history of pushing (NHP) and chronic stroke with a history of pushing (HP). The three axes correspond to the weighting assigned to the body, graviception, and vision, and the data are positioned along the gridlines according to the relative strength of each factor as shown in (C). Purple lines are iso-contour lines for body, blue for graviception, and red for vision. The best-fit biases for each participant are shown as bar graphs in (A) and (B).

Therefore, we quantified individual participants’ sensory weights using a simple three-vector model:^1^
1
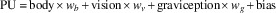
where “body”, “vision”, and “graviception” are unity vectors aligned with the directions of upright signalled by each input. *w*_*b*_, *w*_*v*_, and *w*_*g*_ are the relative weights assigned to each factor and the overall bias captures any constant misalignment of the PU. The model was fit to each participant’s dataset using an established optimization algorithm (the Marquardt-Levenberg technique, see Press et al.^30^) with the constraint that negative weightings were allocated a value of 0. There are just two free weighting parameters since the weights are relative to each other. In order to visualize the distribution of these weights and to compare the five participant types (controls, NHP, HP, NAP, AP), they are plotted as ternary plots in Figure 5. Ternary plots are triangular plots used for plotting sets of three numbers that add up to a constant: in this case 100%. Weightings for controls (group average and confidence intervals [CIs]) and the stroke participant groups (individual scores) are given in Table 1.

##### Control participants

The relative weightings assigned to the sensory inputs contributing to the PU for the age-matched control participants largely cluster towards the bottom right vertex of the ternary plot (Figure 5A). This indicates a relatively large weighting assigned to the body axis and correspondingly smaller weightings assigned to graviception and vision. The mean and 95% CIs for controls were *M*_Body_=62%, CI [41%, 84%], *M*_Vision_=13%, CI [6%, 19%], and *M*_Graviception_=25%, CI [5%, 45%]. Thus, on average control participants appear to assign a moderate to strong weighting of the body to upright, a minor to moderate weighting of graviception, and a minor weighting of vision.

##### Stroke participant groups

The weightings of the chronic stroke participant groups (HP and NHP) are considerably more widely distributed than the controls on the ternary plots (Figure 5B). Weights for each stroke participant are listed individually in Table 1. Mean weights did not differ significantly for any of the three factors (vision, graviception, or body) between HP and NHP participants. The means of the chronic stroke participant group as a whole did not statistically differ from controls. In total, 95% CIs of the mean weightings for the chronic stroke participant group were *Mean*_Body_=45%, CI [27%, 67%], *Mean*_Vision_=18%, CI [9%, 27%], and *Mean*_Graviception_=35%, CI [17%, 54%]. It would appear that in general, the chronic stroke participant group has a pattern of sensory weightings similar to age-matched controls.

Notably, certain individual’s weightings deviated from the general pattern demonstrated by controls and chronic stroke participants, and the weightings of these individuals are informative. For example, all four sub-acute patients demonstrate a 0% weighting for at least one sensory input (see Figure 5B and Table 1). As a very low or negligible weighting of a given input is indicative of poor perceptual reliability,^1^ these scores highlight potential impairment in modality-specific perceptual processing unique to each individual; this can be corroborated by other measures (such as participants reporting the orientation of the long axis of their body^22^). OCHART may be particularly useful in initial detection of these potential perceptual impairments, after which more targeted sensory measures and rehabilitation efforts can be implemented.

## Discussion

At the group level, PU was not differentially affected by changes in body orientation with respect to gravity (graviceptive effect) when comparing (1) controls with individuals with chronic stroke or (2) individuals with chronic stroke with a HP to those without. There were also no significant differences as a result of changing the orientation of the visual cues (visual effect) for either of these group comparisons.

Using group-level analyses alone it may be difficult to characterize existing but subtle patterns in sensory weighting due to the small and heterogeneous group samples. Therefore, we calculated the relative sensory weightings of vision, graviception, and the body for each participant and considered both group-level weightings and individual variations (Figure 5; Table 1). From Figure 5 it is clear that most control participants assign the heaviest weighting to the body, compared with a moderate weighting of graviceptive cues and a minor weighting of visual cues (characterized by scores in the lower right corner of the ternary plot). This distribution of weightings is consistent with previous research on healthy younger adults, although vision was somewhat underemphasized in the control group of this study who had a notably older mean age compared with those tested previously (65 years; vision weighting of 13% here, compared with 25% in Dyde et al.^1^).

Some members of the chronic group showed a similar pattern of weightings to controls (i.e., body>graviception>vision, see for example F and I in Table 1). Other individuals with stroke deviated substantially from this pattern. For example, participants D and O both assigned low weightings to the long axis of their body (see Table 1). This is consistent with other research showing that some individuals with stroke rely more heavily on vision for balance control.^31^ This result is also similar to a higher weighting of vision found in Parkinson’s patients.^32^ A number of other participants’ responses demonstrated a de-emphasis of the body and a greater reliance on graviception for judging upright. However, these patterns of weights were not found in the participants with stroke, and no group-level trends were identified. We argue that individual scores may provide better insights into the specific characteristics of an individual’s perceptions, particularly when weightings deviate dramatically from the typical pattern. Understanding which factors dominate perceptual estimates and which factors are used less by stroke participants compared with the factors used by healthy controls may help to guide customized and targeted rehabilitation interventions. We hypothesized that individuals with AP and a HP would show a bias in their PU, even when all sensory inputs were available and aligned with the body. Bias in the PU under congruent, multisensory conditions (as is typical during everyday life) represents the prevailing estimate of where “up” is. A small but significant leftward bias (2-3°) has been reported among healthy younger individuals.^33^ We did not find any consistent bias in the PU for individuals with a HP when upright, with or without vision, though some showed substantial biases either towards or away from the lesion side (see Figure 4). Notably, the individual with AP showed a significant ~32° ipsilesional bias in his PU under such congruent, aligned conditions, but zero bias in his SVV response. In the following section we consider AP’s responses in more detail, framing these results as an exploratory case study on AP behaviour.

### Characteristics of Active Pushing: A Case Study

The single individual with AP that we tested showed a large and persistent ipsilesional bias regardless of the presence/absence of visual information and was unaffected by tilting the visuals (see visual effect score in Figure 4). AP also showed more reliance on graviceptive inputs (43%) compared with 25% for controls and showed no use of visual inputs (compared with 13% for controls). While he was not influenced by changes in visual cues to upright, changes in his body orientation relative to gravitational upright modulated his PU substantially.

Typical of pushing behaviour, AP’s posture involved a lean in the contralesional direction. The bias we observed in AP’s PU when he was upright was in the ipsilesional direction (to his left). Therefore, it seems likely that the physical behaviour of pushing in this case is a postural compensation for an underlying perceptual error in the opposite direction. Interestingly, AP’s SVV bias was 0° suggesting he did not have any difficulty accurately judging the verticality of a line with respect to gravity. Other authors have suggested that contraversive pushing is driven by an ipsilesional bias in the perceived orientation of the body midline with respect to gravity. That is, when individuals with pushing are positioned upright they feel their body is leaning to the ipsilesional side and they attempt to correct for this by leaning in the other (contralesional) direction.^11^ ^,^ ^20^ Studies investigating SVV in individuals displaying AP have reported, similar to our finding, only minor or no biases in their SVV performance; this has led some to suggest that visual and vestibular cues to upright (which are heavily weighted in the SVV task^1^) may be used normally in individuals with pushing.^11^ ^,^ ^21^ AP’s results are consistent with this theory and the novel contribution that the ipsilesional bias in perceived body midline may be highly robust against visual cues to the contrary. Of course, our observations are based on a single case. More research is needed to confirm whether these results are typical of other individuals with AP, particularly as our findings indicate some individuals with a HP have a bias in SVV that lingers after overt pushing behaviours have resolved.^5^

## Conclusions

When examining the perception of upright it is important to acknowledge that it is informed by at least three factors: vision, graviception, and the internal representation of the orientation of the long axis of the body.^1^ By using OCHART and manipulating the direction of upright signalled by visual and graviceptive inputs relative to the body, we were able to estimate the relative weighting of each of these factors in determining an individual participant’s percept of upright and how these weightings varied across individuals. Though patterns of data can be too variable to generate robust group-level differences, assessing weightings on a case-by-case basis reveals insights into an individual’s potential deficits. We found a large ipsilesional bias in the individual with AP (on average >30°), which was not modulated by visual cues to upright, suggesting that this bias would not necessarily be corrected by providing richer visual cues about the world. In general, the approach of quantifying the weightings of the factors contributing to the perception of upright on an individual basis could have clinical utility in that this information could be used to inform which type of intervention is most likely to be successful.

## Footnotes

a
QUEST staircase initial parameters: Means of 90° to the left/counter-clockwise or 90° to the right/clockwise with respect to the participant’s body midline (the mean is the initial “best guess” of the point of maximal ambiguity, which gives the staircase an idea of what orientations it should test first). SD was 30° (the authors of the QUEST algorithm suggest a liberal standard deviation of the guesses to improve accuracy^29^). Step resolution was 0.5°.
